# Inflammation in Relation to Sarcopenia and Sarcopenic Obesity among Older Adults Living with Chronic Comorbidities: Results from the National Health and Nutrition Examination Survey 1999–2006

**DOI:** 10.3390/nu13113957

**Published:** 2021-11-05

**Authors:** Shama D. Karanth, Caretia Washington, Ting-Yuan D. Cheng, Daohong Zhou, Christiaan Leeuwenburgh, Dejana Braithwaite, Dongyu Zhang

**Affiliations:** 1 Department of Aging and Geriatric Research, University of Florida, Gainesville, FL 32610, USA; shama.karanth@ufl.edu (S.D.K.); cleeuwen@ufl.edu (C.L.); dbraithwaite@ufl.edu (D.B.); 2 University of Florida Health Cancer Center, University of Florida, Gainesville, FL 32610, USA; tingyuan.cheng@ufl.edu (T.-Y.D.C.); zhoudaohong@cop.ufl.edu (D.Z.); 3 College of Medicine, University of Florida, Gainesville, FL 32610, USA; caretia.washingt@ufl.edu; 4 Department of Epidemiology, University of Florida, Gainesville, FL 32610, USA; 5 Department of Pharmacodynamics, College of Pharmacy, University of Florida, Gainesville, FL 32610, USA

**Keywords:** sarcopenia, sarcopenic obesity, inflammation, epidemiology

## Abstract

Loss of muscle mass and waning in muscle strength are common in older adults, and inflammation may play a key role in pathogenesis. This study aimed to examine associations of C-reactive protein (CRP) and systemic immune-inflammation index (SII) with sarcopenia and sarcopenic obesity in older adults with chronic comorbidities. Cross-sectional data from the National Health and Nutrition Examination Survey (1999–2006) were obtained for participants aged ≥60 years. Sarcopenia was defined by a lean mass and body height (males < 7.26 kg/m^2^, females < 5.45 kg/m^2^). Sarcopenic obesity was defined by the concurrent presence of sarcopenia and obesity (defined by relative fat mass). Logistic regression was used to assess the associations of CRP and SII with sarcopenia and sarcopenic obesity. The dose–response relationship was examined via restricted cubic splines. Of the participants (*n* = 2483), 23.1% (*n* = 574) and 7.7% (*n* = 190) had sarcopenia and sarcopenic obesity, respectively. The multivariable logistic regression models suggested a positive association of SII with sarcopenia and sarcopenic obesity, but a positive statistically significant association was not consistently observed for CRP. Dose–response curves suggested similar association patterns for these biomarkers. In clinical practice, measures to prevent sarcopenia and sarcopenic obesity are needed for older vulnerable people with high systemic inflammation.

## 1. Introduction

The aging process is associated with changes in body composition, including the loss of muscle mass, strength, and function [1,2]. Approximately 1–2% loss of muscle mass per year occurs after midlife, and a loss of ~50% of muscle mass by the 8th–9th decades of life has been reported in prior literature [3]. Sarcopenia is characterized by the involuntary loss of skeletal muscle mass due to the aging process [2]. The majority of adults (25–45%) with sarcopenia are those aged 60 years and older [1,4,5], who are more likely to live with a higher burden of comorbidities than younger adults [6]. Sarcopenia with concurrent obesity is referred to as sarcopenic obesity [2]. Several adverse health events are associated with sarcopenia and sarcopenic obesity, including fractures, functional limitation [7], and increased mortality [8,9]. The prevalence of both sarcopenia and sarcopenic obesity increases with age and varies by comorbidities [10], and it has been reported that sarcopenia is highly prevalent (~30%) in individuals with chronic diseases such as cardiovascular disease (CVD), diabetes mellitus, and respiratory disease [11].

Inflammation is defined as an adaptive response that is triggered by deleterious stimuli, such as infection or tissue injury [12,13]. In humans, inflammation can induce muscle protein catabolism (e.g., depletion of body composition, apoptosis of muscle cells, etc.), which increases the risk of sarcopenia and sarcopenic obesity [14]. Older adults—particularly those with chronic comorbidities—usually have a high level of inflammation [2,15]. Thus, describing the burden of sarcopenia and sarcopenic obesity in older vulnerable people, and studying their association with inflammation, can provide relevant evidence to improve healthcare management and prevent muscle depletion in the elderly population. Although several existing epidemiological studies have explored the relationship between inflammation and sarcopenia or sarcopenic obesity, some methodological limitations in these studies make it necessary to re-examine their relationship. For example, several studies had smaller sample sizes [16,17,18], enrolled younger participants [19,20], or did not adjust for the burden of comorbidities [21] in analysis—a critical factor in aging research—making their conclusions less generalizable to older vulnerable adults.

Here, we conducted a cross-sectional analysis of the National Health and Nutrition Examination Survey (NHANES), using data between 1999 and 2006 to examine the association of two available inflammatory biomarkers that can be easily obtained in routine clinical tests—C-reactive protein (CRP), and systemic immune-inflammation index (SII)—for sarcopenia and sarcopenic obesity in older adults with chronic comorbidities. CRP is a non-glycosylated, pentameric protein that is released by the liver in response to inflammatory cytokines [22], while the SII is a novel index developed using the peripheral lymphocyte, neutrophil, and platelet counts, and has been reported to reflect systemic inflammation [23,24,25].

## 2. Methods

### 2.1. Data Source and Study Population

The NHANES is an ongoing cross-sectional survey conducted by the National Center for Health Statistics (NCHS) of the Centers for Disease Control and Prevention; it collects data to measure the medical conditions, health-related behaviors, and nutritional status of U.S. residents. Detailed methods, sample design, and procedure manuals can be found at https://www.cdc.gov/nchs/nhanes/index.htm. Participants with the following characteristics were included for analysis: (1) were aged 60 years or older at interview; (2) had measures of inflammation; (3) had measures of non-imputed values for appendicular lean mass (ALM) (see below); (4) had no missing data of other covariates; and (5) had at least one major type of chronic comorbidity (see below). The final sample for analysis comprised 2483 participants (Supplementary Figure S1). This study was conducted in accordance with the guidelines laid down in the Declaration of Helsinki. All procedures involving research study participants were approved by the NCHS Research Ethics Review Board (Protocol #98-12 and Protocol #2005-06). Due to the de-identified nature of the data analyzed, this study was exempt from review by the University of Florida Institutional Review Board.

### 2.2. Inflammatory Biomarkers

During the interview, the research staff collected blood samples from participants to measure CRP and SII, which were used to reflect levels of inflammation in our study. Specifically, the blood samples were collected from the participants via venipuncture at the mobile examination centers (MECs) according to standard protocols after a 9 h fast. High-sensitivity CRP assays were performed for serum samples via latex-enhanced nephelometry [26]. CRP (mg/L) was categorized into quartiles as follows: <1.2 (reference), 1.2–2.5, 2.6–5.2, and ≥5.3. The SII was calculated using the following equation: (peripheral blood platelet × neutrophil)/lymphocyte counts [25]. Furthermore, SII (×10^9^ cells/L) was categorized into quartiles: <365.7 (reference), 365.7–503.6, 503.7–704.0, and ≥704.1. We chose these two biomarkers as the exposures of interest because they can be easily measured in routine clinical blood tests, making our conclusions more clinically translatable.

### 2.3. Sarcopenia and Sarcopenic Obesity

Between 1999 and 2006, body composition measures were performed using whole-body dual-energy X-ray absorptiometry (DEXA) scans (Hologic Scanner, QDR-4500, Bedford, MA, USA) [27] for adults aged 60 years and older. Appendicular lean mass (ALM), measured by DEXA, and height without shoes, measured by stadiometer, were used to define sarcopenia. Based on criteria used in the European Working Group on Sarcopenia in Older People (EWGSOP), ALM (kg)/height (m)^2^ lower than 7.26 kg/m^2^ was used to define sarcopenia for males, and a value lower than 5.45 kg/m^2^ was used for females [28,29]. Sarcopenic obesity was defined as having sarcopenia and obesity simultaneously. Relative fat mass (RFM)—a sex-specific measure to reflect body fat—was used to define obesity in our study. RFM is a more accurate measure of a whole-body fat percentage than the body mass index (BMI) [30], since BMI does not distinguish between fat mass and non-fat mass. RFM was calculated by the equation (64−(20 × height/waist circumference) +12 × sex) %; in this formula, sex equals 0 for men and 1 for women. Waist circumference was measured by trained examiners, and used the same unit as height. RFM ≥ 30% and RFM ≥ 40% were used to define obesity in males and females, respectively [28].

### 2.4. Covariates

The selection of covariates was based on a priori knowledge regarding the relationships between exposures and outcomes in our study. We included the following self-reported sociodemographic characteristics as covariates: age (60–69, 70–79, and ≥80 years), sex (female vs. male), race (White, Black, or other), educational attainment (high school or less, some college, or graduated from college), and marital status (married vs. not married). Lifestyle behaviors with the potential to impact inflammation and body fat were measured via a self-administered questionnaire [21]. Specifically, participants self-reporting ever smoking at least 100 cigarettes in life were defined as current smokers, or former smokers if they quit. Alcohol consumption (none, ≤1 drink/day, or >1 drink/day) and regular physical activity (no vs. yes) were also included. Regular physical activity was defined as taking part in moderate (only caused light sweating or a slight-to-moderate increase in breathing or heart rate) or vigorous (caused heavy sweating or large increases in breathing or heart rate) activities during the past 30 days. During the interview, the research staff used 24 h dietary recall to measure dietary intake. Macronutrient (carbohydrates, fat, and protein) intake and energy consumption are relevant sources of diet-related inflammation, and their consumption levels are associated with body composition [31,32]. We categorized dietary intake levels into quartiles. RFM was categorized into four levels to reflect low (female: <35%; male: <25%), moderate (female: 35–39.9%; male: 25–29.9%), high (female: 40–44.9%; male: 30–34.9%), and very high (female: ≥45%; male: ≥35%) body fat [33].

We included 12 types of comorbidity for the current study, which were also used to identify eligible study participants. The comorbidities included were CVD (history of heart attack, congestive heart failure, stroke, or coronary heart disease), hypertension, diabetes, chronic renal diseases, chronic respiratory diseases (emphysema, chronic bronchitis), osteoporosis, arthritis, and history of cancer. The burden of comorbidities was further categorized as a binary variable, and living with ≥2 comorbidities was defined as having multimorbidity.

### 2.5. Statistical Analyses

Most analyses used an unweighted approach, because the NHANES weight was generated to reflect distributions in the general U.S. population, which differed from our target population, who were older adults with comorbidities. The participants’ characteristics were summarized overall and by quartiles of inflammatory biomarkers (CRP and SII). Distributions of these variables were described as percentages, and we used χ^2^ tests to examine whether their distributions differed by levels of inflammation. Correlation between CRP and SII was represented by Spearman’s correlation coefficient. The association between inflammatory biomarkers and sarcopenia/sarcopenic obesity was evaluated using logistic regression to estimate odds ratios (ORs) and 95% confidence intervals (CIs). Separate models were performed for CRP and SII. Multivariable logistic regression models for sarcopenia—which treated the lowest quartile of the inflammatory biomarker as the reference—included one biomarker each time, and adjusted for age, sex, race, education, marital status, physical activity, smoking history, alcohol consumption, RFM, multimorbidity, and dietary intake of carbohydrates, total fat, protein, and energy. Models for sarcopenic obesity adjusted for the same variables, except for RFM. Tests for trends were conducted by treating biomarkers as continuous variables. In addition, we corrected for the NHANES sampling weight in the multivariable models, in order to explore whether the effect measures of these biomarkers changed substantially. Furthermore, subgroup analyses were conducted by age (<70 or ≥70 years), sex, and multimorbidity (1 or ≥2 diseases) to explore their interaction with CRP and SII. Old age and high burden of comorbidities indicate a high level of inflammaging [15], while sex was considered because males and females have differential metabolic/nutritional profiles (e.g., anabolism and catabolism regulation of the skeletal muscles), which may have the potential to interact with inflammation [34]. An interaction term between inflammatory biomarkers and these factors was added to the multivariable logistic regression models, and Wald tests were used to evaluate whether the interaction was significant.

Restricted cubic splines were applied for the aforementioned multivariable models, in order to depict a dose–response relationship between biomarkers and sarcopenia/sarcopenic obesity, with the upper limit of the lowest quartile of these biomarkers serving as the reference in the dose–response curve. Because these biomarkers were not normally distributed, a log-transformation was conducted in the dose–response analysis. The test for nonlinearity was conducted by using a likelihood ratio test to compare the model fit using restricted cubic splines with a model fit assuming linearity for the biomarker.

Additionally, we summarized participants’ characteristics by their status of sarcopenia and sarcopenic obesity, and used χ^2^ tests to examine whether their distributions differed by the status of these illnesses. Two sets of sensitivity analyses were conducted to further validate the results obtained from the primary model. To explore whether missing data of covariates could influence the association pattern, we compared ORs from 3 sets of multivariable models to ORs in the primary model. These 3 models included one biomarker each time, and adjusted for the following variables, respectively: (1) age, sex, and race; (2) age, sex, race, marital status, and education; and (3) age, sex, race, dietary components, smoking status, multimorbidity, and alcohol consumption. Then, we explored the association between these biomarkers and sarcopenia/sarcopenic obesity in participants with each individual type of comorbidity, using the same multivariable model as the primary analysis; in this analysis, we treated these biomarkers as binary variables (quartiles 3–4 vs. quartiles 1–2) for sample size consideration. Two-sided *p*-values < 0.05 were considered statistically significant in this study. All statistical analyses were conducted using Stata, version 14.0.

## 3. Results

### 3.1. Characteristics of Participants

Overall, the mean age of participants was 70.2 years (standard deviation (SD) = 7.5); 53.1% of them were younger than 70 years; over half were female (52.0%) or White (56.7%), and approximately two-thirds (64.0%) had more than one comorbidity. Participants with higher CRP levels were more likely to be female or non-White, whereas people with higher SII were more likely to be older or White (Table 1). Participants with sarcopenia or sarcopenic obesity were more likely to be older or White, and sarcopenia was more common in males than in females (Supplementary Table S1). Detailed distributions of other covariates can be found in Table 1 and Supplementary Table S1. There was a significant and positive correlation between CRP and SII (*r* = 0.20, *p* < 0.01).

### 3.2. Association of Sarcopenia with Inflammatory Biomarkers

The overall prevalence of sarcopenia in the study population was 23.1% (95% CI 21.5, 24.8). The prevalence of sarcopenia decreased as CRP increased (<1.2 mg/L: 26.9%, ≥5.3 mg/L: 19.2%). The crude OR of higher CRP levels (≥5.3 vs. <1.2 mg/L) indicated an inverse association (cOR 0.65; 95% CI 0.49, 0.87; *p*-trend < 0.01) (Table 2), but the model suggested a non-significant and positive association after we adjusted for other covariates (aOR 1.29; 95% CI 0.93, 1.80; *p*-trend = 0.06). The weighted analysis also suggested a positive association for CRP, but the effect measures were non-significant. (Table 2).

**Figure 1:**
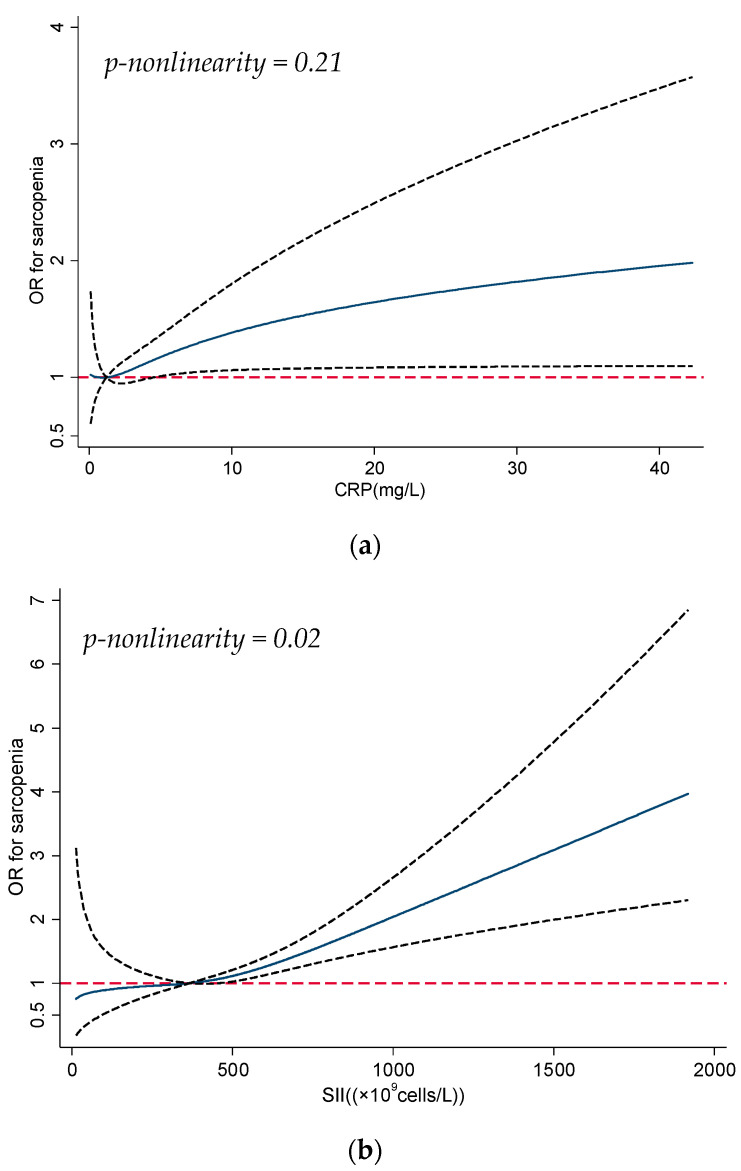
(**a**) Dose–response curve for association between CRP and sarcopenia; the solid line shows the odds ratios (ORs), and the dotted line represents the 95% confidence intervals. CRP = 1.2 mg/L was used as the reference in the curve. Abbreviations—CRP: C-reactive protein. (**b**) Dose–response curve for association between SII and sarcopenia; the solid line shows the odds ratios (ORs), and the dotted line represents the 95% confidence intervals. SII = 365.7 × 10^9^ cells/L was used as the reference in the curve. Abbreviations—SII: systemic immune-inflammation index.

The prevalence of sarcopenia was higher for participants with high SII (≥704.1 × 10^9^ cells/L: 30.8%) compared to those with low SII (<365.7 × 10^9^ cells/L: 19.0%). The unadjusted and adjusted associations between SII and sarcopenia were similar. In the multivariable model, the association between SII and sarcopenia was positively significant (≥704.1 vs. <365.7 × 10^9^ cells/L: aOR 1.90; 95% CI 1.38, 2.62; *p*-trend < 0.01). The weighted analysis also indicated a significant association pattern for SII. The dose–response curves (Figure 1a,b) suggested that the odds of sarcopenia increased with CRP (*p*-nonlinearity = 0.21) and SII (*p*-nonlinearity = 0.02) in a monotonic pattern, although the curve for CRP was non-significant when the level was lower than ~5 mg/L. In subgroup analysis (Table 3), CRP was positively associated with sarcopenia in those aged ≥70 years (*p*-trend = 0.04) or with multimorbidity (*p*-trend = 0.02).

High SII was significantly associated with higher odds of sarcopenia in all subgroups defined by age, sex, and multimorbidity. No significant interaction was identified among these strata. The sensitivity analysis suggested that the effect measures of SII were largely unchanged in models adjusting for different sets of covariates, whereas effect measures of CRP remained inverse if we did not adjust for RFM (Supplementary Table S2a). In the analysis for individual comorbidities (Supplementary Table S2b), the overall association pattern suggested a positive relationship between these biomarkers and sarcopenia/sarcopenic obesity, although some estimates were non-significant because of wide 95% CIs.

### 3.3. Association of Sarcopenic Obesity with Inflammatory Biomarkers

The overall prevalence of sarcopenic obesity in the study population was 7.7% (95% CI 6.6–8.8%). Although the prevalence of sarcopenic obesity increased with CRP levels, the multivariable logistic regression model did not suggest a significant association for CRP (aOR 1.46; 95% CI 0.92, 2.33; *p*-trend = 0.15) (Table 4).

**Figure 2:**
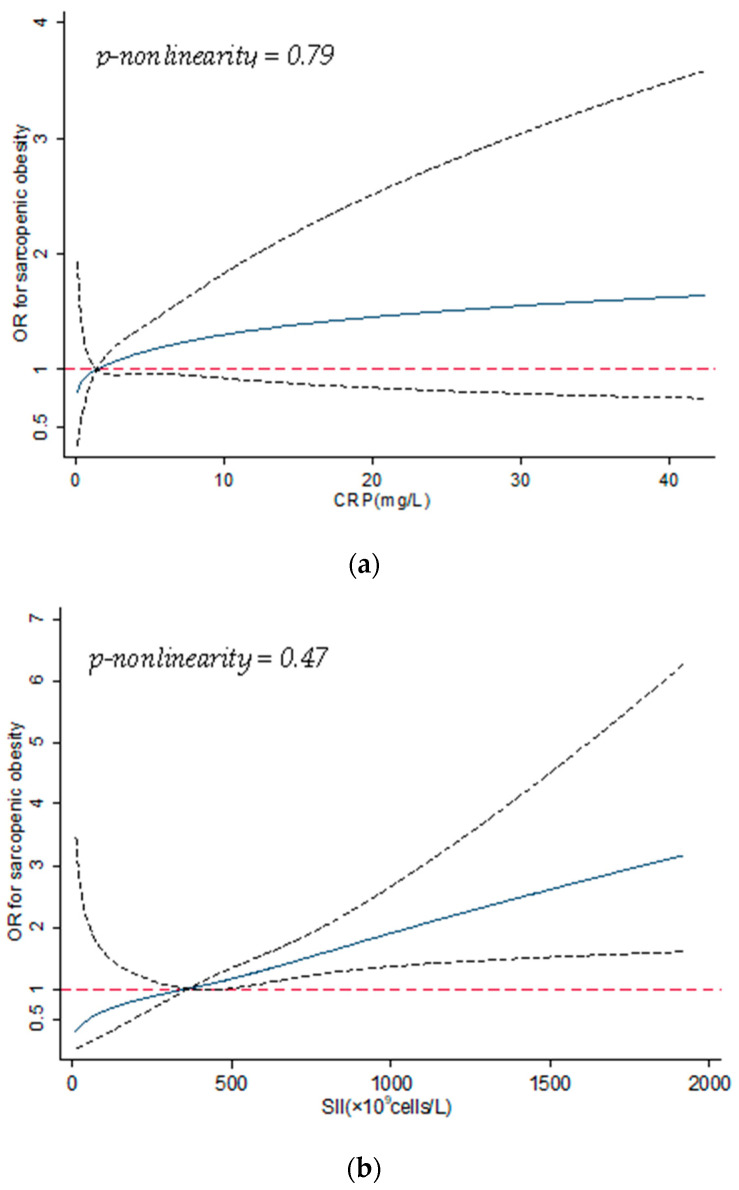
(**a**) Dose–response curve for association between CRP and sarcopenic obesity; the solid line shows the odds ratios (ORs), and the dotted line represents the 95% confidence intervals. CRP = 1.2 mg/L was used as the reference in the curve. Abbreviations—CRP: C-reactive protein. (**b**) Dose–response curve for association between SII and sarcopenic obesity; the solid line shows the odds ratios (ORs), and the dotted line represents the 95% confidence intervals. SII = 365.7 × 10^9^ cells/L was used as the reference in the curve. Abbreviations—SII: systemic immune-inflammation index.

Prevalence of sarcopenic obesity increased with SII; the multivariable logistic regression model indicated a positive and significant association between SII and sarcopenic obesity (aOR = 1.94; 95% CI 1.23, 3.07; *p*-trend < 0.01). The dose–response curves (Figure 2a,b) showed consistent patterns with the multivariable logistic regression models.

Specifically, the curves suggested a positive but non-significant association between CRP and sarcopenic obesity, whereas the pattern was positively significant for SII; neither of these curves indicated nonlinearity. In the subgroup analysis by age, sex, and multimorbidity, no significant effect modification was seen (Table 5).

Based on the sensitivity analysis, effect measures of CRP and SII were largely unchanged in models adjusting for different sets of covariates (Supplementary Table S3a). In the analysis for individual comorbidities, the ORs of SII were all positive, although some of them were non-significant; however, all ORs of CRP were non-significant (Supplementary Table S3b).

## 4. Discussion

In this cross-sectional study that aimed to examine the association of two inflammatory biomarkers with sarcopenia and sarcopenic obesity, we found that sarcopenia and sarcopenic obesity were common in older adults with chronic diseases. Our study suggests that there is a positive association of CRP and SII with sarcopenia and sarcopenic obesity, which indicates that older vulnerable people with high systemic inflammation are more likely to have these conditions compared to counterparts with lower levels of inflammation. However, the results for CRP were not consistently significant.

Interestingly, we found that the association patterns of CRP and SII were slightly different. When investigating sarcopenia, the association of CRP did not become positive (OR > 1) before we adjusted for RFM, whereas this phenomenon was not observed for SII. In our study population, RFM was a negative confounder between CRP and sarcopenia, because it was positively associated with CRP and inversely associated with sarcopenia, suggesting that the direction of ORs of CRP can be flipped if multivariable models do not adjust for RFM. In contrast to SII, statistical significance was not consistently observed for model-adjusted ORs of CRP; moreover, effect sizes of ORs of CRP were less substantial compared to the effect measure obtained for SII. These are unexpected phenomena, for which we do not have a good explanation. One speculation is that their underlying biological heterogeneity (e.g., origins, half-life, etc.) gives these two biomarkers different accuracy in differentiating between people with and without elevated inflammation. Compared to participants with multimorbidity, point estimates of aORs of SII were larger among those without multimorbidity. Larger point estimates of aORs were also seen among the older age group (≥70 years) and females. However, given that the Wald tests did not suggest any statistical significance, we cannot conclude that the relationships between these inflammatory biomarkers and sarcopenia (or sarcopenic obesity) differ by age, sex, or burden of comorbidities.

Our results are consistent with previous studies investigating the association of inflammation with sarcopenia and sarcopenic obesity amongst older adults [7,35,36,37]. A meta-analysis reported that blood CRP levels were significantly higher among patients (mean age > 60 years) with sarcopenia compared to those without sarcopenia (standardized mean difference = 0.51; 95% CI = 0.26, 0.77) [38]. A longitudinal study conducted in the Netherlands reported that high CRP (>6.1 mg/L compared to <1.4 mg/L) was associated with a 2–3-fold greater risk of losing muscle strength [37]. A cross-sectional study conducted in China using data from community-dwelling older adults aged ≥65 years reported that serum high-sensitivity CRP levels were significantly higher in males with sarcopenic obesity (adjusted mean difference = 0.6 mg/L; *p* = 0.036) compared to individuals without sarcopenic obesity [39]. Another cross-sectional study analyzed data from 4224 Chinese adults (mean age: 62.3 years), and reported that high SII was significantly associated with sarcopenia (SII ≥ 406.6 vs. <261.8 10^9^/L; OR = 1.27; 95% CI = 1.03, 1.56) [36]. Our analysis expanded the conclusions from these previous studies by investigating two different biomarkers simultaneously in older adults who are more vulnerable due to the high burden of coexisting chronic diseases. Although physicians use CRP > 10 mg/L to define elevated inflammation in clinical practice, treating CRP as a binary variable, and using this cutoff in the analysis, would induce a compromised power compared to quartiles. Furthermore, using quartiles in a multivariable model ensures that each category has a similar number of observations, thereby reducing random error in analysis.

Several mechanisms may explain the positive association between inflammation and sarcopenia/sarcopenic obesity. First, inflammation is strongly associated with apoptosis [40]. Laboratory evidence suggests that tumor necrosis factor-α (TNF-α)—a biomarker of systemic inflammation—increases with age, and is connected to muscle atrophy and cell loss in rats [41]. The muscle mass is preserved via a balance between protein synthesis and degradation [42], whereas pro-inflammatory mediators induce protein degradation in the skeletal muscle [40]. In addition, prior research suggests that people with high systemic inflammation are more likely to have high fat mass. Schrager et al. investigated 378 men and 493 women aged ≥65 years, and reported that central obesity was significantly associated with increased inflammatory biomarkers (IL-6, CRP) which, in turn, negatively affected the muscle strength and contributed to the development and progression of sarcopenic obesity [43].

Our study has several notable strengths in design and statistical analysis. We investigated two inflammatory biomarkers that can be easily measured in routine clinical tests, enabling us to compare association patterns by these biomarkers and translate the outcomes to health practice. Restricted cubic splines and sensitivity analysis further validated the results obtained in the primary multivariable regression models. Furthermore, since RFM is a sex-specific measure [33], it makes the classification of sarcopenic obesity more precise compared to previous studies that used body mass index or waist circumference for classification [44]. However, there are some limitations to this study. First, as a cross-sectional study, the causal relationship between exposure and outcome could not be evaluated in the analysis. Second, comorbidities were obtained via self-report, which is less accurate compared to review of medical records. Additionally, older adults with multiple comorbidities may receive several different medications to treat their diseases, and these treatments may impact inflammatory biomarkers and body composition [45]; however, the NHANES does not have accurate measures to reflect treatment utilization for these comorbidities, inducing potential residual confounding in analysis. In our analysis, we did not differentiate between specific types of nutrients (e.g., plant vs. animal protein) or exercise (vigorous vs. moderate exercise), which could also induce some residual confounding. As we included participants with at least one chronic disease, our conclusions may be less generalizable to healthier older adults who are living without any comorbidities. Lastly, dietary intake and physical exercise were self-reported measures; hence, there could be some measurement errors associated with the recall.

In conclusion, given the positive association between inflammatory biomarkers and sarcopenia/sarcopenic obesity, geriatricians should consider monitoring these biomarkers in clinical practice for older adults—especially those living with a high burden of comorbidities. Both pharmaceutical and non-pharmaceutical interventions (e.g., diet, exercise, and weight management) that can lower levels of inflammation in the elderly population should be considered as preventive measures to reduce the risk of sarcopenia and sarcopenic obesity. For example, an anti-inflammatory diet has been found to have the potential to prevent sarcopenia; a population-based study among 300 older adults suggested that low dietary inflammatory index—a measure reflecting inflammation related to dietary intake—was associated with a lower prevalence of sarcopenia [46]. As statistical significance was not consistently observed for CRP, future prospective cohort studies should consider measuring CRP longitudinally in order to further explore its relationship with sarcopenia and sarcopenic obesity. Future studies should also consider measuring disease treatments and dietary intake patterns in order to further explore the causal relationship between inflammation and sarcopenia/sarcopenic obesity.

## Figures and Tables

**Table 1:** Participant characteristics by quartiles of inflammatory biomarkers, NHANES 1999–2006 (*n* = 2483).

Variables	Overall	CRP (mg/L)				*p*-Value	SII (×10^9^ cells/L)				*p*-Value
*n* (%)	2483 (100)	<1.2	1.2–2.5	2.6–5.2	≥5.3		<365.7	365.7–503.6	503.7–704.0	≥704.1	
		599 (24.1)	636 (25.6)	619 (24.9)	629 (25.4)		620 (25.0)	623 (25.1)	619 (24.9)	621 (25.0)	
**Ag** **e (years)**											
60–69	1319 (53.1)	319 (53.3)	315 (49.5)	324 (52.3)	361 (57.4)	0.19	357 (57.6)	366 (58.8)	293 (47.4)	303 (48.8)	<0.01
70–79	756 (30.5)	183 (30.5)	204 (32.1)	190 (30.7)	179 (28.5)		181 (29.2)	178 (28.5)	202 (32.6)	195 (31.4)	
≥80	408 (16.4)	97 (16.2)	117 (18.4)	105 (17.0)	89 (14.1)		82 (13.2)	79 (12.7)	124 (20.0)	123 (19.8)	
**Sex**											
Female	1292 (52.0)	247 (41.2)	304 (47.8)	349 (56.4)	392 (62.3)	<0.01	320 (51.6)	315 (50.6)	347 (56.1)	310 (49.9)	0.13
Male	1191 (48.0)	352 (58.8)	332 (52.2)	270 (43.6)	237 (37.7)		300 (48.4)	308 (49.4)	272 (43.9)	311 (50.1)	
**Race**											
White	1408 (56.7)	345 (57.6)	400 (62.9)	343 (55.4)	320 (50.9)	<0.01	272 (43.9)	346 (55.5)	379 (61.2)	411 (66.2)	<0.01
Black	400 (16.1)	82 (13.7)	81 (13.7)	98 (15.3)	139 (22.1)		170 (27.4)	86 (13.8)	78 (12.6)	66 (10.6)	
Other	675 (27.2)	172 (28.7)	155 (24.4)	178 (28.8)	170 (27.0)		178 (28.7)	191 (30.7)	162 (26.2)	144 (23.2)	
**Education**											
High school or less	1555 (62.6)	343 (57.3)	381 (59.9)	410 (66.2)	421 (66.9)	<0.01	396 (63.9)	396 (63.5)	393 (63.5)	370 (59.6)	0.76
Attended college	504 (20.3)	117 (19.5)	129 (20.3)	121 (19.6)	137 (21.8)		123 (19.8)	122 (19.6)	123 (19.9)	136 (21.9)	
Graduated from college	423 (17.1)	139 (23.2)	126 (19.8)	88 (14.2)	71 (11.3)		101 (16.3)	105 (16.9)	103 (16.6)	115 (18.5)	
**Marital status**											
Not married	896 (36.1)	198 (33.1)	203 (31.9)	227 (36.7)	268 (42.6)	<0.01	221 (35.7)	205 (32.9)	224 (36.2)	246 (39.6)	0.11
Married or living with partner	1587 (63.9)	401 (66.9)	433 (68.1)	392 (63.3)	361 (57.4)		399 (64.4)	418 (67.1)	395 (63.8)	375 (60.4)	
**Smoking status**											
Never	1161 (46.8)	304 (50.8)	279 (43.8)	283 (45.7)	295 (46.9)	0.02	311 (50.1)	304 (48.8)	269 (43.5)	277 (44.6)	0.06
Current	310 (12.5)	51 (8.5)	82 (12.8)	86 (13.9)	91 (14.5)		70 (11.3)	63 (10.1)	86 (13.9)	91 (14.6)	
Former	1012 (40.8)	244 (40.7)	275 (43.2)	250 (40.4)	243 (38.6)		239 (38.6)	256 (41.1)	264 (42.6)	253 (40.7)	
**RFM**											
Low	218 (8.8)	91 (15.1)	57 (8.9)	35 (5.7)	35 (5.6)	<0.01	58 (9.3)	50 (8.0)	52 (8.4)	58 (9.3)	0.39
Moderate	672 (27.1)	227 (37.9)	188 (29.6)	141 (22.9)	116 (18.4)		190 (30.7)	170 (27.3)	154 (24.9)	158 (25.4)	
High	1126 (45.4)	228 (38.1)	305 (48.0)	304 (49.1)	289 (45.9)		270 (43.5)	280 (45.0)	286 (46.2)	290 (46.7)	
Very high	467 (18.8)	53 (8.9)	86 (13.5)	139 (22.4)	189 (30.1)		102 (16.5)	123 (19.7)	127 (20.5)	115 (18.5)	
**Regular physical** **activity**											
No	1207 (48.6)	255 (42.6)	286 (45.0)	307 (49.6)	359 (57.1)	<0.01	287 (46.3)	297 (47.7)	309 (49.9)	314 (50.6)	0.40
Yes	1276 (51.4)	344 (57.4)	350 (55.0)	312 (50.4)	270 (42.9)		333 (53.7)	326 (52.3)	310 (50.1)	307 (49.4)	
**Alcohol drinking**											
No	960 (38.6)	219 (36.5)	227 (35.7)	245 (39.6)	269 (42.8)	0.02	266 (42.9)	248 (39.8)	218 (35.2)	228 (36.7)	<0.01
≤1 drink/day	593 (23.9)	162 (27.1)	168 (26.4)	141 (22.8)	122 (19.4)		122 (19.7)	141 (22.6)	190 (30.7)	140 (22.5)	
>1 drink/day	930 (37.5)	218 (36.4)	241 (37.9)	233 (37.6)	238 (37.8)		232 (37.4)	234 (37.6)	211 (34.1)	253 (40.8)	
**Carbohydrate intake (g/day)**											
<153.9	621 (25.0)	111 (18.5)	152 (26.3)	156 (25.2)	202 (32.1)	<0.01	162 (26.1)	158 (25.5)	142 (22.9)	159 (25.6)	0.65
153.9–205.8	593 (23.9)	143 (23.3)	148 (23.3)	157 (25.3)	145 (23.1)		141 (22.7)	160 (25.7)	147 (23.8)	145 (23.4)	
205.9–268.9	643 (25.9)	155 (25.9)	173 (27.2)	160 (25.9)	155 (24.6)		159 (25.7)	156 (25.0)	178 (28.8)	150 (24.1)	
≥269.0	626 (25.2)	190 (31.7)	163 (25.6)	146 (23.6)	127 (20.2)		158 (25.5)	149 (23.9)	152 (24.6)	167 (26.9)	
**Total fat intake (g/day)**											
<42.2	624 (25.1)	134 (22.4)	153 (24.1)	157 (25.4)	180 (28.6)	0.19	162 (26.1)	161 (25.8)	149 (24.1)	152 (24.5)	0.83
42.2–60.1	614 (24.7)	156 (26.0)	143 (22.5)	155 (25.0)	160 (25.5)		152 (24.5)	164 (26.3)	152 (24.6)	146 (23.5)	
60.2–82.9	631 (25.4)	151 (25.2)	170 (26.7)	155 (25.0)	155 (24.6)		158 (25.5)	142 (22.8)	160 (25.8)	171 (27.5)	
≥83.0	614 (24.7)	158 (26.4)	170 (26.7)	152 (24.6)	134 (21.3)		148 (23.9)	156 (25.1)	158 (25.5)	152 (24.5)	
**Protein intake (g/day)**											
< 48.3	617 (24.9)	115 (19.2)	154 (24.2)	165 (26.6)	183 (29.1)	<0.01	142 (22.9)	161 (25.8)	152 (24.6)	162 (26.1)	0.78
48.3–65.4	615 (24.8)	143 (23.9)	139 (21.9)	160 (25.9)	173 (27.5)		150 (24.2)	146 (23.4)	157 (25.4)	162 (26.1)	
65.5–85.1	630 (25.3)	166 (27.7)	165 (25.9)	157 (25.4)	142 (22.6)		158 (25.5)	163 (26.2)	162 (26.2)	147 (23.6)	
≥85.2	621 (25.0)	175 (29.2)	178 (28.0)	137 (22.1)	131 (20.8)		170 (27.4)	153 (24.6)	148 (23.9)	150 (24.2)	
**Energy intake (kcal/day)**											
< 1259.0	622 (25.1)	116 (19.4)	161 (25.3)	159 (25.7)	186 (29.6)	<0.01	154 (24.8)	174 (27.9)	146 (23.6)	148 (23.8)	0.84
1259.0–1654.3	592 (23.8)	145 (24.2)	130 (20.4)	158 (25.5)	159 (25.3)		149 (24.0)	140 (22.5)	146 (23.6)	157 (25.3)	
1654.4–2133.7	641 (25.8)	163 (27.2)	174 (27.4)	151 (24.4)	153 (24.3)		163 (26.2)	154 (24.7)	167 (26.9)	157 (25.3)	
≥2133.8	628 (25.3)	175 (29.2)	171 (26.9)	151 (24.4)	131 (20.8)		154 (24.8)	155 (24.9)	160 (25.9)	159 (25.6)	
Multimorbidity *											
No	894 (36.0)	247 (41.2)	217 (34.1)	226 (36.5)	204 (32.4)	<0.01	238 (38.4)	260 (41.7)	202 (32.6)	194 (31.2)	<0.01
Yes	1589 (64.0)	352 (58.8)	419 (65.9)	393 (63.5)	425 (67.6)		382 (61.6)	363 (58.3)	417 (67.4)	427 (68.8)	
**Respiratory disease ^§^**											
No	2219 (89.4)	559 (93.3)	572 (89.9)	543 (87.7)	545 (86.6)	<0.01	569 (91.8)	572 (91.8)	548 (88.5)	530 (85.4)	<0.01
Yes	264 (10.6)	40 (6.7)	64 (10.1)	76 (12.3)	84 (13.4)		51 (8.2)	51 (8.2)	71 (11.5)	91 (14.6)	
**Hypertension**											
No	906 (36.5)	245 (40.9)	237 (37.3)	219 (35.4)	205 (32.6)	0.02	234 (37.7)	239 (38.4)	233 (37.6)	200 (32.2)	0.09
Yes	1577 (63.5)	354 (59.1)	399 (62.7)	400 (64.6)	424 (67.4)		386 (62.3)	384 (61.6)	386 (62.4)	421 (67.8)	
**CVD ^†^**											
No	1932 (77.8)	454 (75.8)	488 (76.7)	512 (82.7)	478 (76.0)	<0.01	493 (79.5)	494 (79.3)	479 (77.4)	466 (75.0)	0.19
Yes	551 (22.2)	145 (24.2)	148 (23.3)	107 (17.3)	151 (24.0)		127 (20.5)	129 (20.7)	140 (22.6)	155 (25.0)	
**Diabetes**											
No	1949 (78.5)	466 (77.8)	513 (80.7)	474 (76.6)	496 (78.9)	0.34	483 (77.9)	478 (76.7)	500 (80.8)	488 (78.6)	0.36
Yes	534 (21.5)	133 (22.2)	123 (19.3)	145 (23.4)	133 (21.1)		137 (22.1)	145 (23.3)	119 (19.2)	133 (21.4)	
**Chronic kidney** **disease**											
No	2387 (96.1)	586 (97.8)	612 (96.2)	597 (96.5)	592 (94.1)	<0.01	598 (96.5)	606 (97.3)	596 (96.3)	587 (94.5)	0.08
Yes	96 (3.9)	13 (2.2)	24 (3.8)	22 (3.5)	37 (5.9)		22 (3.5)	17 (2.7)	23 (3.7)	34 (5.5)	
**Arthritis**											
No	1159 (46.7)	318 (53.1)	283 (44.5)	290 (46.9)	268 (42.6)	<0.01	306 (49.4)	301 (48.3)	265 (43.8)	287 (46.2)	0.10
Yes	1324 (53.3)	281 (46.9)	353 (55.5)	329 (53.1)	361 (57.4)		314 (50.6)	322 (51.7)	354 (57.2)	334 (53.8)	
**Osteoporosis**											
No	2,163 (87.1)	526 (87.2)	551 (86.6)	535 (86.4)	551 (87.6)	0.85	552 (89.0)	548 (88.0)	526 (85.0)	537 (86.5)	0.16
Yes	320 (12.9)	73 (12.8)	85 (13.4)	84 (13.6)	78 (12.4)		68 (11.0)	75 (12.0)	93 (15.0)	84 (13.5)	
**Cancer**											
No	1989 (80.1)	470 (78.5)	502 (78.9)	499 (80.6)	518 (82.4)	0.30	515 (83.1)	505 (81.1)	482 (77.9)	487 (78.4)	0.08
Yes	494 (19.9)	129 (21.5)	134 (21.1)	120 (19.4)	111 (17.6)		105 (16.9)	118 (18.9)	137 (22.1)	134 (21.6)	

Abbreviations—CRP: C-reactive protein; CVD: cardiovascular diseases; RFM: relative fat mass; SII: systemic immune-inflammation index. Column percentages are reported in the table.* Comorbidities include respiratory diseases, CVD, chronic kidney diseases, osteoporosis, diabetes, arthritis, hypertension, and cancer. ^§^ Respiratory diseases include emphysema and chronic bronchitis. ^†^ CVD (cardiovascular diseases) include heart attack, coronary heart disease, stroke, and congestive heart failure. RFM was categorized based cutoffs validated in prior research to reflect low (female: <35%; male: <25%), moderate (female: 35–39.9%; male: 25–29.9%), high (female: 40–44.9%; male: 30–34.9%), and very high (female: ≥45%; male: ≥35%) body fat.

**Table 2:** Association between inflammatory biomarkers and sarcopenia in older adults with chronic comorbidities, NHANES 1999–2006 (*n* = 2483).

Varibales	No. Sarcopenia /Overall	Prevalence (%) and 95% CI	cOR and 95% CI (*n* = 2483)	aOR and 95% CI ^†^ (*n* = 2483)	aOR and 95% CI ^§^ (*n* = 2483)
**CRP (mg/L)**					
<1.2	161/599	26.9 (23.5, 30.6)	REF	REF	REF
1.2–2.6	151/636	23.7 (20.6, 27.2)	0.85 (0.66 1.10)	1.02 (0.75, 1.38)	0.89 (0.61, 1.33)
2.7–5.2	141/619	22.8 (19.6, 26.3)	0.80 (0.62, 1.04)	1.28 (0.93, 1.76)	1.05 (0.76, 1.46)
≥5.3	121/629	19.2 (16.3, 22.5)	0.65 (0.49, 0.87)	1.29 (0.93, 1.80)	1.44 (0.95, 2.16)
			*p*-trend < 0.01	*p*-trend = 0.06	*p*-trend = 0.07
**SII(×10^9^ cells/L)**					
<365.7	118/620	19.0 (16.1, 22.3)	REF	REF	REF
365.7–503.6	109/623	17.5 (14.7, 20.7)	0.90 (0.68, 1.20)	0.89 (0.64, 1.24)	0.90 (0.59, 1.37)
503.7–704.0	156/619	25.2 (21.9, 28.8)	1.43 (1.09, 1.88)	1.45 (1.05, 2.01)	1.36 (0.84, 2.18)
≥704.1	191/621	30.8 (27.2, 34.5)	1.89 (1.45, 2.46)	1.90 (1.38, 2.62)	2.27 (1.63, 3.15)
			*p*-trend < 0.01	*p*-trend < 0.01	*p*-trend < 0.01

Abbreviations—aOR: adjusted odds ratio; CI: confidence interval; cOR: crude odds ratio; REF: reference; CRP: C-reactive protein; SII: systemic immune-inflammation index. ^†^ The model adjusted for age; sex; race; education; marital status; smoking; relative fat mass; physical activity; alcohol use; dietary intake of carbohydrates, total fat, protein, and energy; and multimorbidity. ^§^ The model adjusted for the same set of covariates, but corrected for the NHANES sampling weight.

**Table 3:** Subgroup analysis for association between inflammatory biomarkers and sarcopenia, NHANES 1999–2006 (*n* = 2483).

Variables		CRP (mg/L)		*p*-Trend		SII (×10^9^ cells/L)		*p*-Trend
		aOR and 95% CI				aOR and 95% CI		
	1.2–2.5 vs. < 1.2	2.6–5.2 vs. <1.2	≥5.3 vs. <1.2		365.7–503.6 vs. <365.7	503.7–704.0 vs. < 365.7	≥704.1 vs. <365.7	
**Age (year)**								
<70 (*n* = 1319)	0.91 (0.57, 1.47)	1.07 (0.66, 1.76)	1.7 (0.70, 1.94)	0.48	0.79 (0.48, 1.32)	1.72 (1.04, 2.85)	1.65 (1.00, 2.72)	<0.01
≥70 (*n* = 1164)	1.21 (0.81, 1.82)	1.57 (1.03, 2.39)	1.46 (0.94, 2.27)	0.04	0.98 (0.62, 1.55)	1.41 (0.91, 2.17)	2.24 (1.45, 3.46)	<0.01
		*p*-interaction = 0.74				*p*-interaction = 0.28		
**Sex**								
Female (*n* = 1292)	0.91 (0.57, 1.45)	1.21 (0.76, 1.95)	1.00 (0.62, 1.62)	0.71	0.74 (0.44, 1.22)	1.45 (0.91, 2.30)	1.66 (1.04, 267)	<0.01
Male (*n* = 1191)	1.22 (0.73, 1.69)	1.29 (0.82, 2.00)	1.40 (0.86, 2.26)	0.13	1.10 (0.69, 1.76)	1.35 (0.84, 2.16)	2.14 (1.36, 3.37)	<0.01
		*p*-interaction = 0.63				*p*-interaction = 0.63		
**Multimorbidity**								
No (*n* = 894)	1.04 (0.61, 1.75)	1.48 (0.86, 2.53)	0.97 (0.54, 1.74)	0.66	1.40 (0.81, 2.39)	1.84 (1.04, 3.27)	2.72 (1.53, 4.84)	<0.01
Yes (*n* = 1589)	1.00 (0.68, 1.48)	1.24 (0.89, 1.87)	1.56 (1.03, 2.36)	0.02	0.64 (0.40, 1.01)	1.29 (0.85, 1.94)	1.66 (1.11, 2.46)	<0.01
		*p*-interaction = 0.32				*p*-interaction = 0.28		

Abbreviations—aOR: adjusted odds ratio; CI: confidence interval; CRP: C-reactive protein; SII: systemic immune-inflammation index. The model was adjusted for the same set of covariates as the primary model, except for those used for stratification.

**Table 4:** Association between inflammatory biomarkers and sarcopenic obesity in older adults with chronic comorbidities, NHANES 1999-2006 (n = 2483).

Variables	No. Sarcopenic Obesity/ Overall	Prevalence (%) and 95% CI	cOR and 95% CI (*n* = 2483)	aOR and 95% CI ^†^ (*n* = 2483)	aOR and 95% CI ^§^ (*n* = 2483)
**CRP (mg/L)**					
<1.2	35/599	5.8 (4.2, 8.0)	REF	REF	REF
1.2–2.5	53/636	8.3 (6.4, 10.8)	1.46 (0.94, 2.27)	1.43 (0.91, 2.26)	1.37 (0.72, 2.61)
2.6–5.2	52/619	8.4 (6.5, 10.9)	1.28 (0.95, 2.30)	1.44 (0.91, 2.28)	1.30 (0.68, 2.48)
≥5.3	50/629	7.9 (6.0, 10.3)	1.39 (0.89, 2.17)	1.46 (0.92, 2.33)	1.61 (0.86, 3.03)
			*p*-trend = 0.19	*p*-trend = 0.15	*p*-trend = 0.19
**SII (×10^9^ cells/L)**					
<365.7	31/620	5.0 (3.5, 7.0)	REF	REF	REF
365.7–503.6	33/623	5.3 (3.8, 7.4)	1.06 (0.64 1.76)	0.97 (0.57, 1.62)	0.99 (0.52, 1.85)
503.7–704.0	57/619	9.2 (7.1, 11.8)	1.92 (1.23, 3.03)	1.58 (0.99, 2.54)	1.34 (0.71, 2.52)
≥704.1	69/621	11.1 (8.9, 13.8)	2.38 (1.53, 3.69)	1.94 (1.23, 3.07)	2.32 (1.32, 4.05)
			*p*-trend < 0.01	*p*-trend < 0.01	*p*-trend < 0.01

Abbreviations—aOR: adjusted odds ratio; CI: confidence interval; cOR: crude odds ratio; REF: reference; CRP: C-reactive protein; SII: systemic immune-inflammation index. ^†^ The model adjusted for age; sex; race; education; marital status; smoking; physical activity; alcohol use; dietary intake of carbohydrates, total fat, protein, and energy; and multimorbidity. ^§^ The model adjusted for the same set of covariates, but corrected for the NHANES sampling weights.

**Table 5:** Subgroup analysis for association between inflammatory biomarkers and sarcopenic obesity, NHANES 1999-2006 (*n* = 2483).

Variables	CRP (mg/L)			*p*-Trend	SII (×10^9^ cells/L)			*p*-Trend
		aOR and 95% CI				aOR and 95% CI		
	1.2–2.5 vs. <1.2	2.6–5.2 vs. <1.2	≥ 5.3 vs. <1.2		365.7–503.6 vs. <365.7	503.7–704.0 vs. <365.7	≥704.1 vs. <365.7	
**Age (year)**								
<70 (*n* = 1319)	1.15 (0.51, 2.61)	1.31 (0.60, 2.88)	1.43 (0.65, 3.13)	0.34	0.59 (0.24, 1.44)	2.01 (0.95,4.25)	1.46 (0.66, 3.20)	0.06
≥70(*n* = 1164)	1.62 (0.93, 2.83)	1.48 (0.84, 2.62)	1.36 (0.75, 2.45)	0.43	1.29 (0.67, 2.48)	1.46 (0.79, 2.69)	2.41 (1.35, 4.30)	<0.01
	*p*-interaction = 0.78					*p*-interaction = 0.08		
**Sex**								
Female (*n* = 1292)	1.66 (0.85, 3.27)	1.37 (0.70, 2.69)	1.27 (0.65, 2.49)	0.78	0.92 (0.43, 1.96)	1.85 (0.94, 3.61)	2.45 (1.27, 4.74)	<0.01
Male (*n* = 1191)	1.30 (0.69, 2.45)	1.58 (0.82, 3.03)	1.62 (0.82, 3.18)	0.13	1.01 (0.49, 2.11)	1.46 (0.73, 2.92)	1.78 (0.92, 3.44)	0.04
	*p*-interaction = 0.69					*p*-interaction = 0.96		
**Multimorbidity**								
No (*n* = 894)	0.96 (0.38, 2.39)	1.63 (0.71, 3.74)	1.30 (0.53, 3.20)	0.34	1.52 (0.56, 4.08)	2.58 (0.98, 6.78)	2.54 (0.97, 6.63)	0.03
Yes (*n* = 1589)	1.68 (0.97, 2.88)	1.52 (0.86, 2.68)	1.61 (0.91, 2.83)	0.18	0.80 (0.43, 1.51)	1.34 (0.77, 2.32)	1.83 (1.07, 3.11)	<0.01
	*p*-interaction = 0.67					*p*-interaction = 0.76		

Abbreviations—aOR: adjusted odds ratio; CI: confidence interval; CRP: C-reactive protein; SII: systemic immune-inflammation index. The model was adjusted for the same set of covariates as the primary model, except for those used for stratification.

## Data Availability

Publicly available datasets were analyzed in this study; these data can be found here: https://www.cdc.gov/nchs/nhanes/index.htm (accessed date: 1 May 2021).

## Supplementary Materials

The following are available online at https://www.mdpi.com/article/10.3390/nu13113957/s1: Figure S1: Flowchart of the study participants’ selection; Table S1: Participant characteristics by sarcopenia and sarcopenic obesity, NHANES 1999–2006; Table S2a: Comparison across models adjusting for different sets of covariates for sarcopenia; Table S2b: Association of inflammatory biomarkers with sarcopenia in older adults with each individual comorbidity; Table S3a: Comparison across models adjusting for different sets of covariates for sarcopenic obesity; Table S3b: Association of inflammatory biomarkers and sarcopenic obesity in older adults with each individual comorbidity.
