# Supplementary material for: Inflammation in Relation to Sarcopenia and Sarcopenic Obesity among Older Adults Living with Chronic Comorbidities: Results from the National Health and Nutrition Examination Survey 1999–2006
Source: Nutrients. 2021 Nov 5;13(11):3957. doi: 10.3390/nu13113957 (PMC8621174; doi:10.3390/nu13113957)
Supplement: Supplementary file 1 [file nutrients-13-03957-s001.zip › nutrients-1422223-supplementary.pdf]

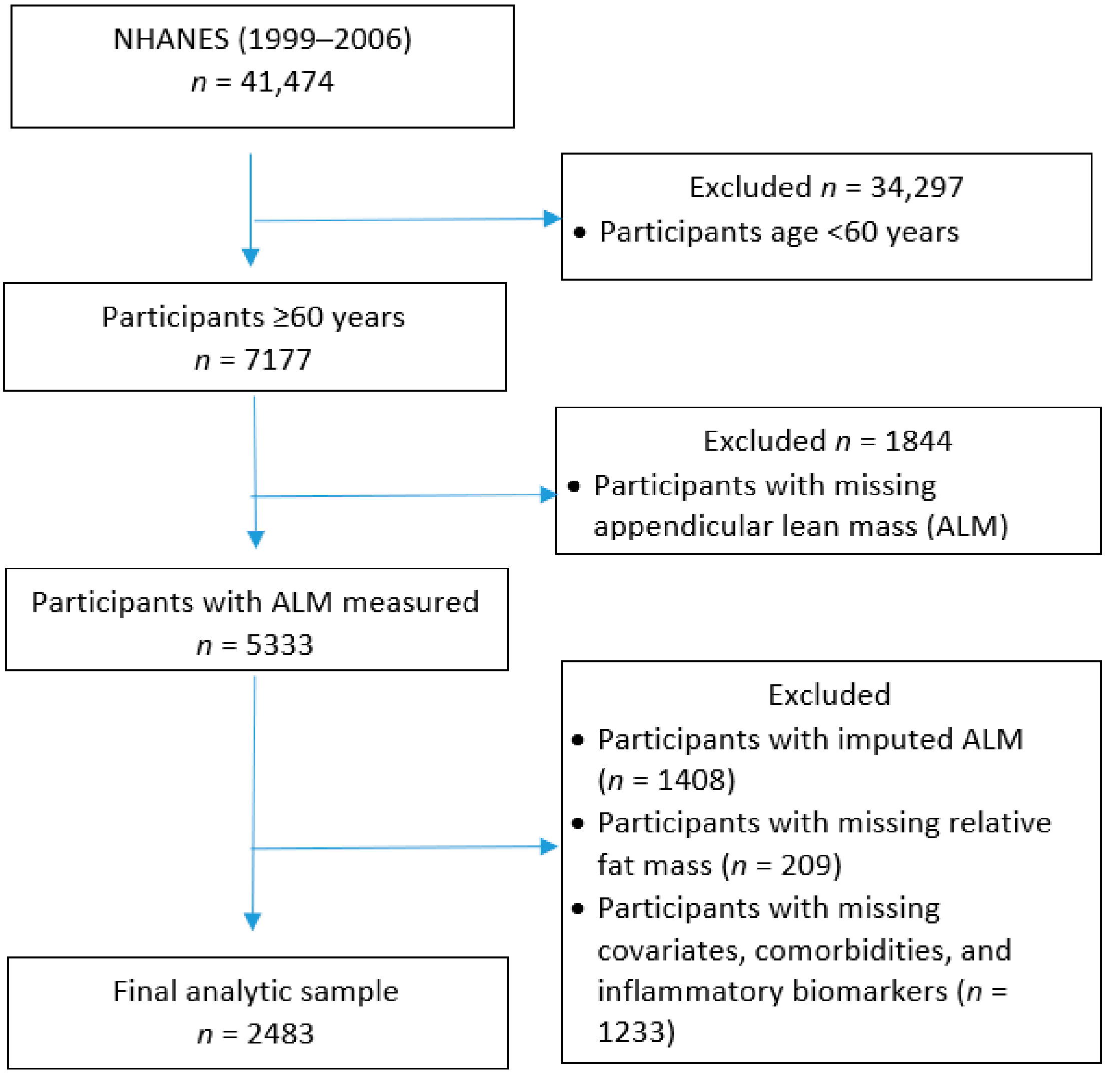

Supplementary Figure S1. Flowchart of the study participants selection. Abbreviations: NHANES: National Health and Nutrition Examination Survey; ALM: Appendicular lean mass.

Supplementary Table S1. Participant characteristics by sarcopenia and sarcopenic obesity, NHANES 1999–2006.

Variables	Sarcopenia		*p*-Value	Sarcopenic Obesity		*p*-Value
	No (*n* = 1909)	Yes (*n* = 574)		No (*n* = 2293)	Yes (*n* = 190)	
**Age (year)**						
60–69	1107 (58.0)	212 (36.9)	<0.01	1259 (54.9)	60 (31.6)	<0.01
70–79	560 (29.3)	196 (34.2)		692 (30.2)	64 (33.7)	
≥80	242 (12.7)	166 (28.9)		342 (14.9)	66 (34.7)	
**Sex**						
Female	1028 (53.9)	264 (46.0)	<0.01	1196 (52.2)	96 (50.5)	0.67
Male	881 (46.1)	310 (54.0)		1097 (47.8)	94 (49.5)	
**Race**						
White	1030 (54.0)	378 (65.9)	<0.01	1288 (56.1)	120 (63.2)	<0.01
Black	363 (19.0)	37 (6.4)		396 (16.3)	4 (2.1)	
**Other**	516 (27.0)	159 (27.7)		609 (26.6)	66 (34.7)	
**Education**						
High school or less	1182 (61.9)	373 (65.0)	0.41	1421 (62.0)	134 (70.5)	0.05
Attended college	395 (20.7)	109 (19.0)		471 (20.5)	33 (17.4)	
Graduated from college	332 (17.4)	92 (16.0)		401 (17.5)	23 (12.1)	
**Marital status**						
Not married	669 (35.0)	227 (39.6)	0.05	814 (35.5)	82 (43.2)	0.035
Married	1240 (65.0)	347 (60.5)		1479 (64.5)	108 (56.8)	
**Smoking status**						
Never	918 (48.1)	243 (42.3)	<0.01	1076 (46.9)	85 (44.7)	0.33
Current	214 (11.2)	96 (16.7)		291 (12.7)	19 (10.0)	
Former	777 (40.7)	235 (41.0)		926 (40.4)	86 (45.3)	
**RFM (%)**						
<35% (female)/<25% (male)	83 (4.6)	135 (23.5)	<0.01	218 (9.5)	0 (0.0)	<0.01
35–39.9% (female)/25–29.9% (male)	423 (22.2)	249 (43.4)		672 (29.3)	0 (0.0)	
40–44.9% (female)/<30–34.9% (male)	946 (49.5)	180 (31.4)		946 (41.2)	180 (94.7)	
≥45% (female)/≥35% (male)	457 (23.9)	10 (1.7)		457 (19.9)	10 (5.3)	
**Regular exercise**						
No	908 (47.6)	299 (51.1)	0.06	1109(48.4)	98 (51.6)	0.39
Yes	1001 (52.4)	275 (47.9)		1184 (51.6)	92 (48.4)	
**Alcohol drinking**						
No	723 (37.9)	237 (41.3)	0.31	879 (38.3)	81 (42.6)	0.49
≤1 drink/day	465 (24.4)	128 (22.3)		552 (24.1)	41 (21.6)	
>1 drink/day	721 (37.8)	209 (36.4)		862 (37.6)	68 (35.8)	
**Carbohydrate intake (g/day)**						
<153.9	471 (24.7)	150 (26.1)	0.59	563 (24.6)	58 (30.5)	0.09
153.9–205.8	448 (23.5)	145 (25.3)		543 (23.7)	50 (26.3)	
205.9–268.9	500 (26.2)	143 (24.9)		597 (26.0)	46 (24.2)	
≥269.0	490 (25.6)	136 (23.7)		590 (25.7)	36 (19.0)	
**Total fat intake (g/day)**						
<42.2	469 (24.6)	155 (27.0)	0.10	567 (24.7)	57 (30.0)	0.26
42.2–60.1	461 (24.1)	153 (26.7)		564 (24.6)	50 (26.3)	
60.2–82.9	486 (25.5)	145 (25.7)		587 (25.6)	44 (23.2)	
≥83.0	493 (25.8)	121 (21.1)		575 (25.1)	39 (20.5)	
**Protein intake (g/day)**						
<48.3	468 (24.5)	149 (26.0)	<0.01	564 (24.6)	53 (27.9)	0.02
48.3–65.4	447 (23.4)	168 (29.3)		556 (24.3)	59 (31.1)	
65.5–85.1	487 (25.5)	143 (24.9)		583 (25.4)	47 (24.5)	
≥85.2	507 (26.6)	114 (19.9)		590 (25.7)	31 (16.3)	
**Energy intake (kcal/day)**						
<1,259.0	470 (24.6)	152 (26.5)	0.10	566 (24.7)	56 (29.5)	0.17
1,259.0-1,654.3	458 (24.0)	134 (23.3)		542 (23.6)	50 (26.3)	
1,654.4-2133.7	478 (25.0)	163 (28.4)		594 (25.9)	47 (24.7)	

≥2133.8	503 (26.4)	125 (21.8)		591 (25.8)	37 (19.5)	
**Multimorbidity***						
No	680 (35.6)	214 (37.3)	0.47	844 (36.8)	50 (26.3)	<0.01
Yes	1229 (64.4)	360 (62.7)		1449 (63.2)	140 (73.7)	
**Respiratory disease§**						
No	1733 (90.8)	486 (84.7)	<0.01	2059 (89.8)	160 (84.2)	0.02
Yes	176 (9.2)	88 (15.3)		234 (10.2)	30 (15.8)	
**Hypertension**						
No	642 (33.6)	264 (46.0)	<0.01	836 (36.5)	70 (36.8)	0.92
Yes	1267 (66.4)	319 (54.0)		1457 (63.5)	120 (63.2)	
**CVD†**						
No	1500 (78.6)	432 (75.3)	0.09	1792 (78.2)	140 (73.7)	0.15
Yes	409 (21.4)	142 (24.7)		501 (21.8)	50 (26.3)	
**Diabetes**						
No	1476 (77.3)	473 (82.4)	<0.01	1805 (78.7)	144 (75.8)	0.35
Yes	433 (22.7)	101 (17.6)		488 (21.3)	46 (24.2)	
**Chronic kidney disease**						
No	1844 (96.6)	543 (94.6)	0.03	2213 (96.5)	174 (91.6)	<0.01
Yes	65 (3.4)	31 (5.4)		80 (3.5)	16 (8.4)	
**Arthritis**						
No	859 (45.0)	300 (52.3)	<0.01	1068 (46.6)	91 (47.9)	0.73
Yes	1050 (55.0)	274 (47.7)		1255 (53.4)	99 (52.1)	
**Osteoporosis**						
No	1676 (87.8)	487 (84.8)	0.06	2000 (87.2)	163 (85.8)	0.57
Yes	233 (12.2)	87 (15.2)		293 (12.8)	27 (14.2)	
**Cancer**						
No	1559 (81.7)	430 (74.9)	<0.01	1844 (80.4)	145 (76.3)	0.17
Yes	350 (18.3)	144 (25.1)		449 (19.6)	45 (23.7)	
Breast cancer	50 (2.6)	21 (3.7)		63 (2.7)	5 (2.6)	
Prostate cancer	61 (3.2)	33 (5.7)		86 (3.8)	8 (4.2)	
Colorectal cancer	32 (1.7)	14 (2.4)		40 (1.7)	6 (3.2)	
Lung cancer	9 (0.5)	5 (0.9)		12 (0.5)	2 (1.1)	
Melanoma	19 (1.0)	8 (1.4)		26 (1.3)	1 (0.5)	

Abbreviations: CRP: C-reactive protein, CVD: cardiovascular diseases, RFM: relative fat mass, SII: systemic immune-inflammation index. Column percentage was reported in the table. * Comorbidities include respiratory diseases, CVD, chronic kidney diseases, osteoporosis, diabetes, arthritis, hypertension, and cancer. § Respiratory disease include emphysema, chronic bronchitis. † CVD, cardiovascular diseases include heart attack, coronary heart disease, stroke, congestive heart failure. RFM was categorized based cutoffs validated in prior research to reflect low (female: <35%, male: <25%), moderate (female: 35–39.9%, male: 25–29.9%), high (female: 40–44.9%, male:30–34.9%), and very high (female: ≥45%, male: ≥35%) body fat.

Supplementary Table S2a. Comparison across models adjusting for different sets of covariates for sarcopenia.

Variables	Model 1 (*n* = 2664)	Model 2 (*n* = 2602)	Model 3 (*n* = 2553)
**CRP (mg/L)**			
<1.2	REF	REF	REF
1.2–2.5	0.81 (0.62, 1.04)	0.80 (0.62, 1.04)	0.78 (0.59, 1.01)
2.6–5.2	0.79 (0.61, 1.03)	0.83 (0.63, 1.07)	0.73 (0.55, 0.96)
≥5.3	0.70 (0.53, 0.92)	0.70 (0.53, 0.92)	0.65 (0.49, 0.86)
	p-trend=0.01	*p*-trend = 0.01	*p*-trend < 0.01
**SII (×10⁹ cells/L)**			
<365.7	REF	REF	REF
365.7–503.6	0.84 (0.63, 1.11)	0.82 (0.61, 1.09)	0.86 (0.64, 1.16)
503.7–704.0	1.11 (0.84, 1.45)	1.14 (0.87, 1.50)	1.16 (0.87, 1.55)
≥704.1	1.57 (1.21, 2.04)	1.52 (1.17, 1.99)	1.56 (1.19, 2.06)
	p-trend<0.01	*p*-trend < 0.01	*p*-trend < 0.01
**Adjusted factors**	Age, sex, and race	Age, sex, race, marital status, and education	Age, sex, race, smoking status, dietary intake (carbohydrate, total fat, protein, energy), alcohol, and multimorbidity

Abbreviations: aOR: adjusted odds ratio, CI: confidence interval, REF: reference, CRP: C-reactive protein, SII: systemic immune-inflammation index. Multimorbidity includes respiratory diseases, CVD, chronic kidney diseases, osteoporosis, diabetes, arthritis, hypertension, and cancer.

Supplementary Table S2b. Association of inflammatory biomarkers with sarcopenia in older adults with each individual comorbidity.

Comorbidity	No. Sarcopenia/Total (%)	aOR and 95% CI CRP	aOR and 95% CI SII
Respiratory disease	88/264 (33.3)	1.44 (0.68, 3.01)	3.54 (1.58, 7.91)
Hypertension	310/1577 (19.7)	1.26 (0.93, 1.72)	1.82 (1.33, 2.48)
CVD	142/551 (25.8)	1.08 (0.66, 1.77)	2.01 (1.23, 3.28)
Diabetes	101/534 (18.9)	1.38 (0.79, 2.42)	1.42 (0.84, 2.42)
Chronic kidney disease	31/96 (32.3)	2.56 (0.39, 16.8)	1.00 (0.14, 6.96)
Arthritis	274/1324 (20.7)	1.45 (1.05, 2.00)	2.31 (1.66, 3.22)
Osteoporosis	87/320 (27.2)	1.38 (0.72, 2.64)	1.49 (0.79, 2.81)
Cancer	144/494 (29.2)	1.49 (0.90, 2.46)	1.96 (1.17, 3.29)

Abbreviations: aOR: adjusted odds ratio, CI: confidence interval, CRP: C-reactive protein, CVD: cardiovascular diseases, SII: systemic immune-inflammation index. The models adjusted for age, sex, race, education, marital status, smoking, relative fat mass, physical activity, alcohol use, dietary intake of carbohydrates, total fat, protein, and energy. In the multivariable model, CRP and SII were treated as binary variables (quartiles 3-4 vs. quartiles 1-2).

Supplementary Table S3a. Comparison across models adjusting for different sets of covariates for sarcopenic obesity.

Variables	Model 1 (n = 2664)	Model 2 (n = 2602)	Model 3 (n = 2553)
**CRP (mg/L)**			
<1.2	REF	REF	REF
1.2–2.5	1.53 (0.99, 2.36)	1.49 (0.95, 2.32)	1.44 (0.92, 2.25)
2.6–5.2	1.58 (1.02, 2.44)	1.60 (1.03, 2.49)	1.38 (0.88, 2.18)
≥5.3	1.65 (1.06, 2.58)	1.60 (1.02, 2.52)	1.46 (0.95, 2.36)
	p-trend = 0.04	p-trend = 0.05	p-trend = 0.13
**SII (×10^9 cells/L)**			
<365.7	REF	REF	REF
365.7–503.6	0.97 (0.60, 1.57)	0.96 (0.58, 1.58)	1.05 (0.64, 1.74)
503.7–704.0	1.48 (0.95, 2.30)	1.53 (0.98, 2.41)	1.64 (1.03, 2.60)
≥704.1	1.82 (1.19, 2.81)	1.85 (1.20, 2.88)	1.92 (1.23, 3.01)
	p-trend < 0.01	p-trend < 0.01	p-trend < 0.01
**Adjusted factors**	Age, sex and race	Age, sex, race, marital status, and education	Age, sex, race, smoking status, dietary intake (carbohydrate, total fat, protein, energy), alcohol and multimorbidity

Abbreviations: aOR: adjusted odds ratio, CI: confidence interval, REF: reference Multimorbidity includes respiratory diseases, CVD, chronic kidney diseases, osteoporosis, diabetes, arthritis, hypertension, and cancer.

Supplementary Table S3b. Association of inflammatory biomarkers and sarcopenic obesity in older adults with each individual comorbidity.

Comorbidity	No. Sarcopenic Obesity/Total (%)	aOR and 95% CI CRP	aOR and 95 CI SII
Respiratory disease	30/264 (11.4)	1.99 (0.80, 5.00)	3.90 (1.32, 11.58)
Hypertension	120/1577 (7.6)	1.17 (0.79, 1.74)	2.05(1.33, 3.13)
CVD	50/551 (9.1)	0.76 (0.40, 1.47)	2.30 (1.16, 4.56)
Diabetes	46/534 (8.6)	1.33 (0.69, 2.64)	1.27 (0.65, 2.45)
Chronic kidney disease	16/96 (16.7)	4.06 (0.63, 26.2)	1.38 (0.31, 6.15)
Arthritis	99/1324 (7.5)	1.06 (0.69, 1.63)	2.39 (1.49, 3.84)
Osteoporosis	27/320 (8.4)	0.69 (0.28, 1.71)	2.17 (0.81, 5.8)
Cancer	45/494 (9.1)	1.03 (0.50, 2.11)	1.60 (0.61, 3.38)

Abbreviations: aOR: adjusted odds ratio, CI: confidence interval, CRP: C-reactive protein, CVD: cardiovascular diseases, SII: systemic immune-inflammation index. The models adjusted for age, sex, race, education, marital status, smoking, physical activity, alcohol use, dietary intake of carbohydrates, total fat, protein, and energy. In the multivariable model, CRP and SII were treated as binary variables (quartiles 3-4 vs. quartiles 1-2).
